# A System of Practical Surgery

**Published:** 1839-01

**Authors:** 


Art. IX.—*A System of Practical Surgery: with numerous explanatory Plates, the Drawings after Nature.* By John Lizars. Part I.—*Edinburgh*, 1838. 8vo. pp. 220.

It is painful to us to be called on to pass judgment—as, we are sorry to say, we not unfrequently are—on the indifferent or bad productions of men every way respectable and estimable, as well in their professional as private relations. We are in this predicament while noticing the work whose title we have transcribed above. We cannot think how so sensible a man as Mr. Lizars could have deemed a book like this to be needed in the present state of our literature; or how, so thinking, a surgeon of his acknowledged excellence should have written so bad a book.

In his dedication, Mr. Lizars tells the president and fellows of the Royal College of Surgeons of Edinburgh that, ever since he has been engaged in the duties of the professorship, he has "felt the want of a book containing each surgical disease, and the corresponding operation, succinctly and correctly delineated; so as to be a useful guide to the student and remembrancer to the practitioner." Our judgment of it is, therefore, to be founded on its fitness for the intended object; but, as our time will not allow us to comment upon its whole contents, we shall select for examination a single subject, and that the first that presents itself; viz. *Luxations.* At page 170, the student is guided to a correct understanding of "luxation of shoulder-joint," (Mr. Lizars having a peculiar habit of omitting definite or indefinite articles, according to some rule which we have vainly attempted to discover.) Under this head no mention is made of the *causes* of dislocations of the shoulder-joint; the student being left to discover, by some other guide, whether a direct blow on the shoulder-joint, or force transmitted through the humerus from either the elbow or hand, is more likely to dislocate the bone. Surely, Mr. Lizars must be well aware of the practical importance of studying causes in dislocations of bones. The *diagnosis* of luxations of the shoulder-joint is treated with great simplicity: here it is.

"When the head of the bone of the arm is forced into the axilla, there is marked depression under the acromion scapulæ, the arm is semi-bent, the patient supports it with the sound one, and rests it on the thigh or pelvis, and generally complains of a good deal of pain. The surgeon cannot raise it to a right angle with the body, and the attempt aggravates the sufferings. In a day or two sometimes considerable œdematous swelling supervenes." (p. 170.)

Mr. Lizars says not a word about any change in the length of the limb, or about the separation of the elbow from the side, and the change in the axis of the bone; or about the head of the bone being capable of being felt in the axilla; or about flattening of the deltoid muscle; or about the increased difficulty of diagnosis in cases of luxated shoulder-joint which are of long standing, &c. &c. One would think from this that the diagnosis of luxation of the shoulder-joint were always an easy task. After giving one mode of reduction for recent cases, and a method with pulleys for cases of long standing, the curiosity of the student is sated by the satisfactory instruction that "there are various other modes of reduction not necessary to be particularized." On the question of reduction after dislocation of some standing, there is literally nothing, excepting age which is referred to, to guide the student; and not a single word is there about *differential diagnosis.* "Simple luxation, with fracture of the humerus," says Mr. L., "either at its cervix or middle, now and then occurs; in which case we should first endeavour to reduce the dislocation, and then to set the fracture." Mr. Lizars does not at all state that it is first necessary to find out the coexistence of fracture and dislocation.

We are sorry to say that the literary merits of this volume are on a par with its surgical. The following is one instance of the author's contempt for an antecedent:

"Counter-irritants are rubefacients, blisters, moxas, and setons. Rubefacients are designed to redden the skin, or, in other words, to cause a determination to the surface, to remove the inflamed condition of *those* seated in the diseased organ." (p. 19.)

In speaking of the effect of bloodletting, Mr. Lizars says that "the patient feels *nauseous* and sick even to vomiting:" and the following are a few among many of the singular infelicities of style with which this volume abounds:

"The treatment of this affection generally yields to the application of nitrate of silver or potass to the wound." (p. 44.)—"A gland in the groin, before incising, should be allowed to have thoroughly suppurated." (p. 48.)—"Chronic frequently supervenes after the acute, as for instance in ophthalmia, and is also occasionally an idiopathic disease." (p. 13.)—"*Bruit de soufle.*" (p. 92.)—"All acute inflammations are liable to become chronic; but there is a chronic state which begins *à priori,* as, for instance, that which precedes lumbar and other chronic abscesses, and the formation of tumours." (p. 80.)—"Fomentations and poultices are more useful than cold saturnine lotions, in all instances of acute inflammation; *heat being more natural and manageable than cold.* Caloric subdues the action of the nervous and circulating systems by relaxation and exhaustion, *and can be procured either in cold or hot weather.*" &c. (p. 18.)

It may be thought that we have been somewhat harsh in our comments upon Mr. Lizars' performance: but, when our surgical literature abounds in treatises of great merit, and it is said that a comprehensive *system* is needed, it becomes our duty to expose such glaring defects as those which we have noticed, and which we might easily multiply a hundred-fold. The book has no claim whatsoever to be regarded as a "System of Practical Surgery:" it doubtless contains many valuable facts, and its illustrations are not without utility; but on such grounds as these we cannot recommend it to our readers.

